# Viruses and thyroiditis: an update

**DOI:** 10.1186/1743-422X-6-5

**Published:** 2009-01-12

**Authors:** Rachel Desailloud, Didier Hober

**Affiliations:** 1 Laboratoire de Virologie/UPRES EA3610 Faculté de Médecine, Université Lille 2, CHRU Lille, Centre de Biologie/Pathologie et Parc Eurasanté, 59037 Lille, France; 2 Service d'Endocrinologie-Diabétologie-Nutrition, CHU Amiens, 80054 Amiens, France

## Abstract

Viral infections are frequently cited as a major environmental factor involved in subacute thyroiditis and autoimmune thyroid diseases This review examines the data related to the role of viruses in the development of thyroiditis.

Our research has been focused on human data. We have reviewed virological data for each type of thyroiditis at different levels of evidence; epidemiological data, serological data or research on circulating viruses, direct evidence of thyroid tissue infection. Interpretation of epidemiological and serological data must be cautious as they don't prove that this pathogen is responsible for the disease. However, direct evidence of the presence of viruses or their components in the organ are available for retroviruses (HFV) and mumps in subacute thyroiditis, for retroviruses (HTLV-1, HFV, HIV and SV40) in Graves's disease and for HTLV-1, enterovirus, rubella, mumps virus, HSV, EBV and parvovirus in Hashimoto's thyroiditis. However, it remains to determine whether they are responsible for thyroid diseases or whether they are just innocent bystanders. Further studies are needed to clarify the relationship between viruses and thyroid diseases, in order to develop new strategies for prevention and/or treatment.

## Background

Viral infections are frequently cited as a major environmental factor implicated in subacute thyroiditis and autoimmune thyroid diseases [1]. The term thyroiditis encompasses a heterogeneous group of disorders characterized by some form of thyroid inflammation. To categorize the different forms of thyroiditis, most thyroidologists use the following terms: i/Infectious thyroiditis (which includes all forms of infection, other than viral); ii/Subacute thyroiditis (also called subacute granulomatous thyroiditis and which causes acute illness with severe thyroid pain); iii/Autoimmune thyroid disease which includes Hashimoto's thyroiditis (and painless thyroiditis also known as silent thyroiditis or subacute lymphocytic thyroiditis which is considered as a variant form of chronic Hashimoto's thyroiditis) and Grave's disease; iiii/Riedel's thyroiditis which is a very rare disease characterized by extensive fibrosis and mononuclear infiltration.

This review examines the data related to the possible role of viruses in the development of thyroiditis. We have added thyroid lymphoma to the section on Riedel's thyroiditis as both diseases are known complications of autoimmune thyroiditis. Our research has been focused on human data but we used some animal data in order to emphasize some mechanisms and to support such a possibility in humans. We have reviewed virological data at different levels of evidence; epidemiological data, serological data which have been associated with research into circulating viruses and direct evidence of thyroid tissue infection.

### I/Subacute and autoimmune thyroiditis: a viral infection of the thyroid gland?

First defined by De Quervain, subacute thyroiditis is a self-limited inflammatory disorder of the thyroid gland. The disease is most prevalent in females, usually characterized by a sudden onset of neck pain and thyrotoxicosis. Clinically the disease has several characteristics typical of viral infections including a typical viral prodrome with myalgias, malaise and fatigue. Recurrent subacute thyroiditis has been reported [2]. The follicles are often infiltrated, resulting in disrupted basement membrane and rupture of the follicles. The thyroid injury in subacute thyroiditis is thought to be the result of cytolytic T-cell recognition of viral and cell antigens present in an appropriate complex [3].

#### I-A/Epidemiological evidence

The first descriptions showed a tendency for the disease to follow upper respiratory tract infections or sore throats, which explained why a viral infection has most often been implicated as the cause. Clusters of the disease have been reported during outbreaks of viral infection [4]. Onset of the disease are observed between June and September and this seasonal distribution is almost identical to that of established infections due to some enteroviruses (Echovirus, Coxsackievirus A and B), suggesting that enterovirus infections might be responsible for a large proportion of cases [5,6].

An association between subacute thyroiditis and HLA B35 is noted in all ethnic groups tested [7] and two-thirds of patients manifest HLA-B35. Familial occurrence of subacute thyroiditis [8] and recurrence during the course of time [9] are associated with HLA B35. Thus, the onset of subacute thyroiditis is genetically influenced and it appears that subacute thyroiditis might occur through a susceptibility to viral infection in genetically predisposed individuals. HLA-B35 has been reported to be correlated with chronic active hepatitis, with hepatitis B [10], with rapid progression of AIDS [11] and with the T lymphocyte responses against human parvovirus B19 [12]. Recently, the medical records of 852 patients with subacute thyroiditis have been studied. The significant seasonal clusters of subacute thyroiditis during summer to early autumn was confirmed. According to the authors, "the history of patients showed no obvious association with virus infection". Unfortunately, no data on infections are available in the paper [2].

#### I-B/Virological data

Virus-like particles were first demonstrated in the follicular epithelium of a patient suffering from subacute thyroiditis. Judging from the size, it was thought to be influenza or mumps virus [13], which was concordant with an increased frequency of antibodies to the influenza B virus in patients with thyrotoxicosis [14]. The same year, in five out of 28 patients with subacute thyroiditis, a cytopathic virus was isolated by coculturing patient samples with susceptible cell lines[15]. The agent was later studied by electron microscopy and classified as a paramyxovirus [16]. Subsequently, the agent was reanalyzed by immunofluorescence and electron microscopy and was reclassified as a foamy virus [17]. However, the implication of foamy virus has not been confirmed: a more comprehensive study using different techniques demonstrated no association between foamy-virus infection and thyroiditis in 19 patients [18]. Moreover the expression of HFV gag proteins had not been found by indirect immunofluorescence [19]. As part of a larger study investigating the prevalence of foamy-virus infection in humans, 59 patients with thyroid disorders, including 28 with Quervain's thyroiditis, were analyzed by different techniques including PCR. Again, there was no prevalence of foamy virus infection [20]. The origin of the foamy-virus-like agent in the original publications remains unclear, but because the more comprehensive study was unable to detect foamy-virus infection in de Quervain patients, it is highly unlikely that it is a causative agent of this condition [21].

Some cases could be due to the mumps virus. Subacute thyroiditis has occurred in epidemic form: patients with subacute thyroiditis diagnosed during a mumps epidemic were found to have circulating anti-mumps antibodies even without clinical evidence of mumps [22]. High titers of mumps antibodies have been found in some patients with subacute thyroiditis, and occasionally parotitis or orchitis, usual in mumps, were associated with thyroiditis [23]. In favor of thyroid infection is the fact that in two patients out of 11 with subacute thyroiditis diagnosed during a mumps epidemic, the mumps virus was cultured from thyroid tissue obtained at biopsy [22].

Enteroviruses have been suspected. Patients with subacute thyroiditis, who had no clinical evidence of viral disease, demonstrated increases by at least four times in viral antibodies. These viral antibodies included antibodies to mumps virus, but also coxsackie, adenovirus and influenzae. Coxsackie viral antibodies were the most commonly found, and the changes in their titers most closely approximated the course of the disease [24]. In a case report, thyroiditis was attributed to enterovirus: IgM and IgG were found at a quadruple titer against coxsackievirus B4 whereas no other antibodies were found against other coxsackies, echoviruses or mumps [25]. In 27 consecutive patients with subacute thyroiditis, antibody tests, virus isolation and antigen detection were negative. Enterovirus RNA was not detected by RT-PCR neither in blood samples nor in the thyroid tissue in the fine-needle aspiration samples. Common respiratory viruses were also screened. There was no evidence of viral infections, except one patient who had acute CMV infection[26].

Case reports have implicated – CMV in an infant with acute infection and – EBV in an adult female because of positivity for Epstein-Barr virus-specific antibodies and in a 3-year-old girl suffering from infectious mononucleosis because of the presence of EBV DNA both in plasma and leukocytes [27-29]. However, when thyroid specimens of nine patients obtained by fine-needle aspiration biopsy were examined, no EBV or CMV DNA was detected [30]. Serum virus-specific antibodies to measles, rubella, mumps, type I herpes, chicken pox, human parvovirus B19 and CMV were found in 10 patients during the course of illness. In spite of the presence of IgG to each virus in more than 70% of patients, changes in the IgG titers were observed for those to measles, rubella, chicken pox or CMV in 4 patients [30]. In an adult female, subacute thyroiditis was diagnosed one month after acute infection suggesting that rubella virus could also be implicated [31].

Viral antibody titers to common respiratory tract viruses are often elevated. Since the titers fall promptly and are not increased during recurrence [9] and since multiple viral antibodies may appear in the same patient, the elevation could be an anamnestic response due to the inflammatory condition [32].

Although the search for a viral cause is usually unrewarding, it appears that the thyroid could respond with thyroiditis after invasion by a variety of different viruses and that no single agent is likely to be causative in the syndrome of subacute thyroiditis.

### II/Involment of viral infection in autoimmune thyroid diseases (AITD)

The autoimmune thyroid diseases (AITD) are frequent [33,34]. and include Hashimoto's thyroiditis and Graves' disease. Both disease are characterized by lymphocytic infiltration and the presence of serum anti-thyroperoxydase antibody (TPOAb) and/or anti-thyroglobulin antibody (TgAb) for Hashimoto's thyroiditis and TSH receptor autoantibodies (TSHR-Ab) for Graves' disease.

The mechanisms by which infection may induce an autoimmune response are many, and this makes infections an attractive hypothesis for disease initiation [35,36]. Paradoxically, infections may enhance AITD but may also be protective. Indeed, the hygiene hypothesis implies that the immune system is educated by multiple exposures to different infections allowing it to control autoimmune responses better. Thus, improved living standards associated with decreased exposure to infections are associated with an increased risk of autoimmune disease and the lower socio-economic groups have a reduced prevalence of thyroid autoantibodies [37,38].

However, specific infections could be a triggering factor to disease initiation by liberating antigens (via cell destruction or apoptosis), by forming altered antigens or causing molecular mimicry, by cytokine and chemokine secretion, by inducing aberrant HLA-DR expression and Toll-Like Receptor (TLR) activation. TLRs are a family of cell surface receptors which protect mammals from pathogenic organisms, such as viruses, and are present on non-immune cells including thyrocytes [39]. Moreover, TLR3 recognizes double-stranded (ds) RNA, assumed to be released by viral killing of cells. The dsRNA binding to TLR3, mimicked in vitro by incubation with polyinosine-polycytidylic acid [Poly (I:C)], leads not only to the induction of inflammatory responses but also to the development of antigen-specific adaptive immunity [40]. Then, Hashimoto's thyroiditis has been grouped with insulitis and type-1 diabetes, colitis, and atherosclerosis as an autoimmune and inflammatory disease associated with TLR3/4 overexpression, which is in favor of environmental pathogens [39].

In order to give a comprehensive review, virological data on Hashimoto's thyroiditis and Graves' disease are exposed together in the text but are summarized in independent tables (see tables 1, 2, 3) for each disease and classified in terms of their levels of evidence of infection of the thyroid tissue: epidemiological, serological (or circulating viral genome) and molecular.

**Table 1:** Evidence for infection in subacute thyroiditis.

**in favour of infection**	**references**	**not in favour of infection**	**references**
**Levels of data: Epidemiological**			
distribution of disease during outbreaks of viral infection	[4,22]	no obvious association with virus infection	[2,9,32]
seasonal distribution from June to September	[2,5,6]		
**Serological and/or circulating viral genome**			
mumps virus	[22-24]	mumps virus	[30]
coxsackievirus	[24,25,134]	enterovirus	[26]
adenovirus	[24]	HSV-1,	[30]
EBV	[27,28]	parvovirus B19	[30]
measles, chicken pox, CMV	[30]		
influenzae	[14,24]		
rubella	[30,31]		
CMV	[26,29]		
**Direct evidence of infection**			
human foamy virus	[17]	human foamy birus	[18-20]
mumps	[22]	enterovirus	[26]
		CMV and EBV	[30]

**Table 2:** Evidence for infection in Hashimoto's autoimmune thyroiditis

**in favour of infection**	**references**	**not in favour of infection**	**references**
**Levels of data: Epidemiological**			
antithyroid antibodies following subacute thyroiditis	[32,46]	euthyroidism: nonspecific autoimmune response ?	[32,46]
unknown antithyroid antibodies following subacute thyroiditis	[45]		
seasonality of month of birth	[44]		
HTLV-1	[52,53]	SARS: central hypothyroidism	[115]
HIV	[65]	HIV	[67,68]
non-HIV retrovirus	[69]		
congenital rubella	[88,89,91]	congenital rubella	[90]
HCV, HBV	[107,108,110]	HCV	[111,112]
enterovirus infection during pregnancy	[120]	measles-mumps-rubella vaccination	[51]
**Serological and/or circulating viral genome**			
HTLV-1	[54-58,60,137]	HIAP-1	[78]
congenital and acquired rubella	[88-90,92-94]		
EBV	[99,100]		
Parvovirus	[104]		
**Direct evidence of infection**			
HTLV-1	[59]	HFV	[19]
rubella	[87]	CMV	[97]
HSV	[97]	Enterovirus: RNA detected in various thyroid disease	[119]
Parvoviru	[103]		
EBV	[132]		

**Table 3:** Evidence for infection in Grave's disease

**in favour of infection**	**references**	**not in favour of infection**	**references**
**Levels of data: Epidemiological**			
seasonality of month of birth	[44]		
higher diagnosis and relapse rate in spring and summer	[41,42]		
geographical distribution	[43]		
antibodies or disease onset following subacute thyroiditis	[47-50]	nonspecific response to the inflammatory rection ?	[32]
HTLV1	[62]		
HIV	[65]	lack of anti-thyroid antibodies before the beginning of HAART	[75]
**Serological and/or circulating viral genome**			
HTLV1	[57,60-62]		
HIAP-1	[78]		
HFV	[81,84]	HFV	[20,82,83]
parvovirus	[106]	Enterovirus	[121]
HHV6, HHV7	[101]		
**Direct evidence of infection**			
HTLV-1	[64]		
HIV-1	[70]	SV40	[72,73]
		HIAP-1	[79]
HFV	[19,80]	HFV	[83]
SV40	[85]	HSV, CMV	[97]

#### II-A/Epidemiological data

##### II-A-1/Temporal and geographical considerations

Seasonal trends, possibly related to epidemic infections, have been described in the diagnosis or relapse of Graves' disease with higher rates in spring and summer [41,42]. Geographical differences have also been described in England in the incidence of Grave's disease which could be an indirect sign of environmental factors [43].

More surprinsigly, month of birth was studied in 664 patients with Hashimoto's hypothyroidism and in 359 patients with Graves' hyperthyroidism. Patients had a distinct pattern of distribution for month of birth compared with the general population. These differences point towards a a seasonal viral infection as the initial trigger in the perinatal period, the clinical disease resulting from further specific damage over time [44].

##### II-A-2/Subacute thyroiditis: a trigger of thyroid autoimmunity?

Damage to the thyroid in subacute thyroiditis, which is thought to be a virus-associated syndrome, might release normally sequestered antigens, inducing an immune response. Unknown autoantibodies are found in patients with subacute thyroiditis, and a higher prevalence of thyroid autoantibodies after a mean follow-up interval of 4 years but at low titers has been observed [45,46]. However, thyroid autoantibodies appear at low titer only, often transiently and characterized autoimmune pathologies of the thyroid do not usually occur [32]. These autoimmune phenomena could represent a nonspecific response to the inflammatory release of thyroid antigens rather than a specific autoimmune disease. Rare cases of Hashimoto's thyroiditis have been reported but several cases of the occurrence of Graves' disease after subacute thyroiditis have been published [47-49]. To examine whether subacute thyroiditis triggers TSH receptor antibody, 1697 patients with subacute thyroiditis were tested. Antibodies were found positive in 2% of patients but hyperthyroidism was not always present and some of the patients recovered from thyroid dysfunction without treatment. Therefore, subacute thyroiditis could trigger autoreactive B cells to produce TSH receptor antibodies [50].

##### II-A-3/What about vaccination?

Previous natural infection or vaccination against measles and/or mumps seemed to have an inhibitory effect on the development of thyroid autoantibodies. No evidence was found, that measles-mumps-rubella vaccination may trigger autoimmunity: neither the prevalence nor the levels of antibodies changed 3 months after vaccination [51].

#### II-B/Specific virus data

##### II-B-1/Retrovirus

###### II-B-1-a/Human T lymphotrophic virus-1 (HTLV-1)

HTLV-1 is a human retrovirus highly endemic in the Caribbean islands, Central Africa and south-west Japan.

####### II-B-1-a1/Hashimoto's thyroiditis

Human T lymphotrophic virus-1 has been associated with various autoimmune disorders, including Hashimoto's thyroiditis in patients with HTLV1-associated myelopathy/tropical spastic paraparesis [52,53]. Two patients who developed Hashimoto's thyroiditis, proven with biopsy, were HTLV-I carriers but had no myelopathy/tropical spastic paraparesis [54]. A case-control study was then conducted to determine the frequency of HTLV-I seropositivity among patients with Hashimoto's thyroiditis and the frequency of Hashimoto's thyroiditis in patients with HTLV-I-associated myelopathy/tropical spastic paraparesis. The frequency is significantly higher in the two groups than in the general population [55]. A high prevalence of positivity for thyroid autoantibodies (TPOAb and/or TgAb) and hypothyroidism have also been described in the adult T-cell leukaemia patients and the HTLV-I carriers [56]. In prospective studies in blood donors, the frequency of anti-thyroid antibodies tended to be higher in donors with anti-HTLV-I antibody. HTLV-I and HTLV-II proviral load are significantly higher in the peripheral blood of patients with Hashimoto's thyroiditis than in asymptomatic HTLV carriers [57,58]. Thyroid tissues from two patients with Hashimoto's thyroiditis were examined for the presence of HTLV-I. The virus envelope protein and signals for the mRNA were detected in many of the thyrocytes from one of the patients, by immunohistochemistry and in-situ hybridization respectively. PCR-Southern blotting revealed the presence of HTLV-I DNA although no virus particles were found by electron microscopy. The present findings suggest that infection of thyroid tissue with HTLV-I is possible [59]. An association between HTLV-I infection and autoimmune thyroiditis may be then strongly suspected.

####### II-B-1-a2/Graves' disease (GD)

Serum HTLV-I antibody is found in 6% of patients with GD, 7% of patients with chronic thyroiditis, and 2% of patients with nodular goiter[60]. Beside the fact that anti-HLTV-I antibody and proviral DNA is detected in peripheral lymphocytes of patients with GD [60,61], proviral load in HTLV-I-infected patients with GD, as observed in Hashimoto's thyroiditis, is significantly higher than in asymptomatic HTLV-I carriers [57]. GD and HTLV-I infection seem to be interacting and resulting in onset of uveitis [61,62]. Indeed, 5% of HTLV-I-positive patients with GD developed uveitis, whereas none of the HTLV-I negative patients with GD nor HTLV-I-positive patients with chronic thyroiditis or nodular goiter developed uveitis [60]. The provirus load was significantly higher in the uveitis patients with GD than in those without GD [63]. As in Hashimoto's thyroididtis, HTLV-I infectivity in thyroid was proven: HTLV-I DNA was detected by polymerase chain reaction in the thyroid tissue of an HTLV-I-infected male who was successively afflicted with GD followed by uveitis. HTLV-I was isolated from thyroid tissue by coculture with peripheral blood lymphocytes [64].

#### II-B-1-b/Human Immunodeficiency Virus (HIV)

##### II-B-1-b1/Hashimoto's thyroiditis

Autoimmune diseases in HIV/AIDS have been reported with an array of autoantibodies including anti-thyroglobulin and anti-thyroid peroxidase [65].

A high prevalence of subclinical hypothyroidism with 3.5% to 12.2% has been described in patients receiving HAART [66]. A cross-sectional multicenter study was done to determine the prevalence of and risk factors for hypothyroidism in HIV-infected patients. Of the 350 HIV-infected patients studied; 16% had hypothyroidism, 2.6% had overt hypothyroidism, 6.6% had subclinical hypothyroidism, and 6.8% had a low level of free T4. The prevalence of subclinical hypothyroidism was higher among HIV-infected men. A nested case-control study was conducted which compared hypothyroid and euthyroid patients. Only receipt of stavudine and low CD4 cell count were associated with hypothyroidism [67]. Thyroid dysfunction seemed, therefore, to be due to medication and not to autoimmunity. To confirm these data, 22 HIV+ hypothyroid patients and 22 HIV+ euthyroid controls receiving highly active anti-retroviral therapy were included in an additional study. No goiter or anti-thyroid antibodies were detected. Thus, in our experience, HIV is not a cause of autoimmune thyroiditis [68]. Discordant data have been published about these risk factors. Mechanisms and screening of patients is discussed in a recent review [66].

Some individuals possess antibodies that react to HIV-1 Western blot proteins in patterns different from HIV infection diagnostic. Autoimmune thyroiditis is more frequent in patients exhibiting these indeterminate HIV-1 Western blots in comparison with a control cohort of HIV-negative blots. These data suggested that patients infected with non-HIV retrovirus could develop thyroid autoimmunity [69].

##### II-B-1-b2/Graves' disease (GD)

Autoimmune diseases in HIV/AIDS that have been reported include GD [65]. Several hypotheses have been elaborated. Using Southern blot analysis, specific integration of exogenous sequences homologous to HIV-1 gag region was only found in genomic DNA of thyrocytes from patients with GD and not in normal thyrocytes. These findings suggested that retrovirus-like sequences could be associated with thyroid autoimmunity [70]. High reverse transcriptase activity which resembled that demonstrated in retroviruses has been observed in thyroid tissue extracts obtained by surgery from patients with GD. The reverse transcriptase existed in the thyroid tissue as a complex, with endogenous template RNA, and the activity was confirmed not to be due to other DNA polymerases. In a permissive genetic and immunological environment, retroviral DNA integrated into genomic DNA could then participate to the onset of GD [71]. However, in a study using sets of primer pairs designed to cover the whole span of the HIV-1 gag region, neither Southern blot hybridization nor PCR gave positive signals in any of the samples examined [72] as confirmed in another study [73]. Homology between the HIV-I Nef protein and the human TSHR has been suggested [74]. However, a retrospective analysis of serum samples of a patient with GD revealed lack of anti-thyroid autoantibodies before the beginning of antiretroviral treatment[75].

Despite many attempts, results to date remain inconclusive concerning a direct role of HIV in the onset of GD but a special mechanism has been observed – the immune system recovery. De novo diagnoses of thyroid disease were identified between 1996 and 2002 in seven HIV treatment centers. Patients were diagnosed as clinical case entities and not discovered through thyroid function test screening. GD was diagnosed in 15 out of 17 patients diagnosed with AITD. One patient developed hashithyrotoxicosis and another, hypothyroidism. AITD patients were more likely than controls to be severely compromised at baseline and to experience greater CD4 increments following HAART. Regulatory T lymphocytes (Treg) appear to be important in suppressing autoimmune reactions. It is possible that a relative deficiency of such cells explains the appearance of GD during immune system recovery [75,76].

#### II-B-1-c/Human Intracisternal A-type particles type 1 (HIAP1)

Intracisternal A-type particles (IAPs) are defective retroviruses that assemble and bud at the membranes of the endoplasmic reticulum, where they remain as immature particles consisting of uncleaved polyproteins antigenically related to HIV [77]. Serum antibodies against HIAP-I are detectable in 85% of GD patients compared to only 1.9% of controls. They are absent in patients with Hashimoto's thyroiditis as well as other forms of non-autoimmune thyroid disease. A genetically determined immunological susceptibility has been demonstrated: the class II HLA status allows interactions with HIAP-I exposure and this interaction could be a predisposing factor in the pathogenesis of GD [78]. Intracisternal A-type particles have been reported in H9 cells co-cultured with homogenates of salivary glands obtained from patients with Sjögren syndrome and with synovial fluid of patients with rheumatoid arthritis. However, no HIAP-1 particles are detected by electron microscopy in the H9 cells co-cultured with thyroid preparations of GD. These data call into question the involvement of HIAP-1 in the etiopathogenesis of Graves' disease [79].

#### II-B-1-d/Human Foamy Virus (HFV)

Human foamy virus (HFV) is a member of the retroviral family of Spumaretroviridae. Three retroviral structural proteins of HFV – gag, pol and env – can be identified by indirect immunofluorescence.

##### II-B-1-d1/Hashimoto's thyroiditis

From the thyroids of five patients with Hashimoto's disease, four were negative for the structural protein and one showed a single small focus of anti-gag antibody reactivity [19].

##### II-B-1-d2/Graves' disease

Contrary to what has been found in Hashimoto's thyroiditis, the expression of HFV gag proteins has been demonstrated by indirect immunofluorescence on the epithelial cells of seven out of seven thyroid glands of patients with GD whereas it was negative in 9 subacute thyroiditis and 2 normal glands. The retrobulbar tissue of 1 Graves' disease patient with malignant exophthalmus revealed also positive staining with anti-gag antibodies in fibroblasts and fat cells [19,80]. In a search for spumaretrovirus infection markers, a group of 29 patients with GD and 23 controls were studied. A positive signal with a spumaretrovirus-specific genomic probe was found in DNA extracted from peripheral blood lymphocytes in 10 patients and spumaretrovirus related sequences were detected by PCR in the DNA of 19 patients. All 23 control subjects were negative. These results strongly suggest the existence of an association between GD and the presence of spumaretrovirus related infection markers[81]. Other studies failed to detect the presence of antibodies by several immunodetection techniques and foamy virus DNA in peripheral blood lymphocytes [20,82] and the presence of the spumaretrovirus gag region sequence was not statistically significant in DNA extracted from the peripheral blood leukocytes and thyroid tissue of 81 patients with GD and of 66 controls [83]. Nevertheless, the nature of the HFV-related sequences identified in the genomes of healthy individuals and the GD patients appeared to be different. Three regions of HFV-related sequences were amplified in 29% of the HFV-positive patients, while no samples in the control group amplified all three regions. This suggests that these sequences may be used as a tool for screening for HFV in GD patients [84].

These studies provide no evidence for a causative role for HFV in GD. However, the data do represent the possibility that HFV-like sequences may be implicated and this is a possibility especially in some geographically distinct populations [21].

#### II-B-1e/Simian virus (SV40)

Simian virus 40 (SV40) is a polyomavirus that is found in both monkeys and humans. Like other polyomaviruses, SV40 is a DNA virus that has the potential to cause tumors but most often persists as a latent infection. In a study dedicated to thyroid tumors, SV40 sequences were also investigated in GD thyroid specimens, normal thyroid tissues, and peripheral blood mononuclear cells of healthy donors. Specific SV40 large T antigen sequences were detected, by PCR and filter hybridization, in human thyroid tissues from GD patients, with a frequency of 20% compared with a frequency of 10% of control normal thyroid tissues from patients affected by multinodular goiter [85].

### II-B-2/Rubella virus (German measles)

Thyroid disorders in patients with congenital rubella were first reported in 1975 [86]. Infection of thyroid tissue by rubella was demonstrated in a case of congenital rubella with Hashimoto's thyroiditis: immunofluorescent studies of thyroid tissue demonstrated staining for rubella virus antigen [87]. Thyroid autoantibodies, anti-TPO or anti-Tg antibodies have been found more frequently in patients with congenital rubella syndrome than in controls [88,89]. These studies are old and a recent study has shown that humoral autoimmunity was not so frequent. In 37 subjects affected by or exposed to rubella during fetal life, one patient had diabetes, four patients had clinical hypothyroidism and five patients were positive for TPOAb at the time of the examination [90]. However, in an Australian study, the prevalence of thyroid disorders, as well as diabetes and early menopause, was higher in subjects with congenital rubella (studied 60 years after their intrauterine infection) than the general population. It is worthy of note that 41% of the subjects had undetectable levels of rubella antibodies [91]. Since most reports have shown no evidence of active rubella infection at the time of thyroid dysfunction, the mechanisms proposed for thyroid dysfunction are destruction of thyroid cells by local persistent rubella virus infection, precipitation of an autoimmune reaction, or both [92-96].

### II-B-3/Herpesviridae

The Herpesviridae are a large family of DNA viruses that share a common structure and a common characteristic which is latent and re-occurring infections. Herpesviridae can cause lytic infections.

#### II-B-3a/Herpes Simplex Virus (HSV) and Cytomegalovirus (CMV)

Thyroid tissue specimens were obtained postoperatively from four patients with multinodular goiter and 18 patients with AITD (GD and Hashimoto thyroiditis). Herpesviridae DNA was detected using PCR-based assays. Herpesviridae DNA has been more frequently detected in AITD tissue specimens than in tissue specimens of multinodular goiters. No statistically significant differences were observed concerning the specific strains HSV1, HSV2, HSV 6 or HSV7. No CMV DNA was isolated from any tissue specimen [97].

#### II-B-3b/Epstein Barr Virus (EBV)

EBV infection is known to be involved in tumoral diseases such as lymphoma but also in autoimmune diseases, such as multiple sclerosis, rheumatoid arthritis and systemic lupus erythematosus [98]. Antibodies against EBV viral capsid antigen (IgG-VCA) and antibodies against early antigen (IgG-EA-D/DR) have been more often found in thyroiditis than in controls [99]. What is unusual is that EBV may induce anti-T3 antibodies. Acute EBV infection with severe primary hypothyroidism was described in a 16-year old female patient. She had high low FT4 and low FT3 but discordant elevated total T3. Later, 34 patients with EBV infection were tested for thyroid hormone levels. Five patients with acute EBV and one with previous infection had total T3 values above the mean which was due to anti-T3 antibodies [100].

#### II-B-3c/Human Herpes Virus (HHV)

HHV are ubiquitous, tissue tropism widespread. HHV6 and HHV7 circulating DNA was searched in sixty Graves disease patients paired to 60 controls. Both viruses infection increased the risk for Graves disease especially HHV7 that was significantly more frequent among patients (64.6%) than in controls (38.7%). Patients 72TP53 Pro/Pro variants (inherited diminished TP53 apoptotic function) had 5 times more chance to develop GD and almost three times more chance to be infected by HHV7 which is consistent with interaction between genetics and viral infection in Graves disease physiopathology[101].

### II-B-4/Parvovirus

The B19 virus belongs to the Parvoviridae family of small DNA viruses. Parvovirus B19 is known for causing a childhood exanthem but it has also been associated with autoimmune diseases: autoimmune neutropenia, thrombocytopenia, hemolytic anemia and rheumatoid arthritis [102]. Recently, a few studies have suggested the association of parvovirus infection with thyroiditis.

#### II-B-4a/Hashimoto's thyroiditis

Intrathyroidal persistence of human parvovirus B19 DNA with PCR has been detected for the first time in a patient with Hashimoto's thyroiditis. The cell types responsible for the B19 DNA persistence are not determined and immune cells infiltrating the thyroid may be the source of B19 DNA. However, the possibility that thyroid epithelial cells harbor B19 DNA cannot be excluded [103].

Serum samples from 73 children and adolescents with Hashimoto's thyroiditis and from 73 age-matched controls have been analyzed for the presence of specific antibodies. No differences are observed. But Parvovirus B19 DNA, indicating recent B19-infection, is detectable more frequently in patients and a negative correlation exists with disease duration. There is strong evidence that acute parvovirus B19 infections are involved in the pathogenesis of some cases of Hashimoto's thyroiditis[104].

Parvovirus may also be present in the brain. Some authors hypothesize that parvovirus B19 is a common human pathogen which could explain the association between mental disorders and thyroid diseases because of its ability to infect the brain and to induce autoimmunity. This hypothesis is based on the fact that they found in patients with bipolar disorder both a thyroid disorder and brain B19 infection [105].

#### II-B-4b/Graves' disease

A woman, whose son had an episode of exanthem two weeks previously, was infected with parvovirus and suffered successively GD, type-1 diabetes and rheumatic polyarthritis. Serological tests showed IgM antibodies to human parvovirus B19, but no IgM antibodies to cytomegalovirus, Epstein Barr virus, rubella, measles, or Coxsackie viruses. Anti-TSH receptor antibody was positive. Parvovirus viral protein 1 was detected in her bone marrow samples but no analysis was done on thyroid tissue [106].

### II-B-5/Viral Hepatitis C and B (HCV and HBV)

Discordant data are published about hepatitis. Thyroid involvement may be regarded as the most frequent alteration in HCV positive patients and is more frequent than in HVB. The prevalence of abnormally high levels of anti-thyroid antibodies varied markedly, ranging from 2% to 48% and subclinical hypothyroidism has been observed in 2 to 9% of patients with chronic hepatitis C [107,108]. In a retrospective cohort study which included 146,394 patients infected with HCV (individuals with human immunodeficiency virus were excluded) and 572,293 patients uninfected with HCV, thyroiditis risk was slightly increased, but there was no analysis of treatment [109]. The prevalence of autoimmune thyroid disease in patients with HCV differs from that in patients with the hepatitis B virus (HBV) before, at the end of, and 6 months after stopping treatment with IFN-alpha. Positive levels of TPOAb and TgAb were found in 20% and 11% of patients with HCV compared with 5% and 3% of patients with HBV, respectively. At the end of IFN-alpha therapy, thyroid gland dysfunction was more prevalent in patients with HCV (12%) compared with those with HBV (3%), with TSH levels significantly higher in the HCV group [110]. Other authors don't find an association between hepatitis C virus and thyroid autoimmunity [111,112]. Thyroid autoimmunity may be a cytokine-induced disease in susceptible patients. Indeed, the incidence is much greater in females and positive anti-TPOAb patients prior to the initiation of therapy. According Marazuela, thyroid dysfunction secondary to interferon is reversible after discontinuation of therapy [113], which is discordant with Fernandez' data [110]. Variable geographic distribution has also shown that genetic or environmental influences could be implicated [114]. On the whole, the distinctive role of the virus itself or antiviral treatment remains to be clarified. Abnormalities in thyroid function should be included among the complications of HCV syndrome and patients should be periodically screened for thyroid involvement in order to identify patients in need of treatment as quoted in a recent review [107].

### II-B-6/SARS coronavirus (SARS-CoV)

A substantial number of patients with SARS have shown abnormalities in thyroid function. As SARS is a disease known to cause multiple organ injury, it has been supposed that SARS could have a harmful effect on the thyroid gland. However, low serum triiodothyronine and thyroxine levels associated with decreased TSH concentration are in favor of central hypothyroidism induced by hypophysitis or by hypothalamic dysfunction [115].

### II-B-7/Enterovirus

#### II-B-7-a/Hashimoto's thyroiditis

Enteroviruses play a role in immune-mediated pathological processes, such as chronic myositis and chronic dilated cardiomyopathy [116]. Epidemiologic and prospective studies have provided a body of arguments that strongly suggest the role of enteroviruses in type-1 diabetes in which, interestingly, AITD is frequently observed [117,118]. Recently, we have shown that EV-RNA can be detected by real-time PCR in thyroid tissue from patients with various thyroid diseases, but no relationship between the presence of EV-RNA and thyroiditis, lymphocytic infiltration or the presence of circulating TPOAb was found. Although the patients in our series are certainly different from patients with classic Hashimoto's thyroiditis, which are rarely treated surgically, our results suggest that the presence of EV-RNA in thyroid tissue is not associated with autoimmune thyroiditis. It's worthy to note that EV-RNA was detected in one patient with a normal thyroid [119]. Maternal enterovirus infection during pregnancy has been linked to thyroiditis in children. Sera taken at delivery from mothers whose children subsequently developed AITD was analyzed for antibodies against enterovirus, and compared with a control group. Of the mothers whose children developed AITD, 16% were enterovirus IgM-positive, compared with 7% in the control group which was not statistically different. However, the age at diagnosis of AITD was significantly lower in the group of children with IgM-positive mothers compared with children with IgM-negative mothers. Also hypothyroidism was significantly more frequent in the IgM-positive group, with no child in the IgM-negative group [120].

#### II-B-7-b/Graves' disease

Patients with recent onset of Graves' hyperthyroidism (about two months before blood sample collection) have been investigated in regard to enterovirus infection. A nested PCR reaction with primers of the enterovirus genome was employed on blood samples but all were negative for RNA of the enterovirus group [121].

#### II-C/Viral involvement in the etiology of hypothyroidism: animal models data

Reovirus infection of a neonatal mouse can induce thyroiditis and thyroid autoimmunity. Mice infected with reovirus type 1 develop a thyroiditis characterized by focal destruction of acinar tissue, infiltration of the thyroid by inflammatory cells, and production of autoantibodies directed against thyroglobulin and thyroid microsomes [122]. The segment of the reovirus type 1 genome responsible for the induction of autoantibodies to thyroglobulin encodes a polypeptide that binds to surface receptors and determines the tissue tropism of the virus [123].

An endogenous retrovirus (ev 22) was found to be expressed in obese-strain (OS) chickens but not in healthy normal strains. Ev 22 is inherited autosomally in a dominant manner. The OS chickens develop a hereditary spontaneous autoimmune thyroiditis characterized histologically by lymphocytic infiltration of the thyroid gland. Thyroiditis is associated with obesity and hyperlipidemia. A similar thyroiditis has also been induced in normal chickens by retroviral infection [124].

Lymphocytic choriomeningitis virus (LCMV) can persist in the thyroid gland of three strains of mice neonatally infected with the virus. Furthermore, the virus that was shown to persist mainly in the thyroid epithelial cells in which thyroglobulin is synthesized induced a reduction in the level of thyroglobulin messenger RNA and circulating thyroid hormones, but there was no thyroid cell destruction. Then persistent, apparently benign virus infection with LCMV, can be induced in the thyroid of mice and this infection induces thyroid dysfunction. This alteration in thyroid homeostasis is not caused to the thyroid by autoantibodies. Moreover, despite infection of the thyroid gland, neither necrosis nor inflammation occurs [125].

Animals models show that a typical autoimmune thyroiditis can be induced by a direct viral infection but also by an inherited retrovirus infection. These models also show that thyroid dysfunction can occur without inflammation or antithyroid autoantibodies. Further studies are then needed in humans to explore the role of viruses in the pathogenesis of thyroid dysfunctions.

### III/Lymphomas and Riedel's thyroiditis

Lymphomas and Riedel's thyroiditis are rare disorders but both can occur in association with Hashimoto's thyroiditis.

#### III-A/Riedel's thyroiditis

Fibrous thyroiditis, also known as Riedel's thyroiditis, is characterized by extensive fibrosis and mononuclear infiltration that extends into adjacent tissues. It may consist in a primary fibrosing disorder or in the local involvement of a multifocal fibrosclerosis. The etiology of Riedel's thyroiditis is not known. It can occur in association with Hashimoto's thyroiditis [126]. As this disease is rare, the literature is scarce.

Two cases of Riedel's thyroiditis onset have been reported after a subacute thyroiditis, which is thought, as already said, to be a viral induced disaese. Two women, first diagnosed with sub-acute thyroiditis, developed an enlargement of the thyroid gland and symptoms of compression eight months and three years later, respectively. Post-operative histopathologic evaluation showed Riedel's thyroiditis characteristics associated with sub-acute thyroiditis [127,128].

Only one case report of infection has been reported in international literature. A 36-year old woman had of long-term fever associated with a biologic inflammatory syndrome which was reported as due to EBV infection because of a positive EBV serology. TSH concentration, levels of TPOAb and thyrocalcitonin were normal. There was a dramatic improvement after thyroidectomy with normalization of inflammatory parameters. The role of EBV infection in the process of this unusual form of Riedel's thyroiditis was suspected [129].

#### III-B/Lymphomas

Thyroid lymphomas are nearly always of the non-Hodgkin's type. Hodgkin's lymphoma of the thyroid is exceedingly rare. Preexisting chronic autoimmune thyroiditis is the only known risk factor for primary thyroid lymphoma, and is present in about one-half of patients [130].

### III-B-1/Epstein Barr Virus (EBV)

Epstein-Barr virus (EBV) is found in many lymphomas. The clinicopathological characteristics in the Hong Kong Chinese population and the presence of EBV in thyroid lymphomas were analyzed by reviewing data collected over three decades. EBV gene expression by in-situ hybridization and immunohistochemistry were performed. Primary thyroid lymphomas were found in 23 patients and secondary lymphomas were found in 9 patients. EBV messenger RNAs were detected in one primary and one secondary thyroid lymphoma [131].

One study explored the association of EBV with thyroid lymphoma (TL) and with chronic lymphocytic thyroiditis (CLTH) which is known to play an important role in the development of TL. Thirty cases with TL and 28 with CLTH were studied for presence or absence of EBV genome in the lesions, using the polymerase chain reaction (PCR) and the in-situ hybridization method. EBV genomes were detected by PCR in one CLTH and two TL. In-situ hybridization revealed positive signals in the nucleus of lymphoma cells, which also expressed latent membrane protein-1 [132].

EBV-related mRNA presence was investigated in 32 cases of malignant lymphoma of the thyroid by in-situ hybridization and immunohistochemistry. EBV-encoded small RNA were detected in three cases [133]. These findings indicate that EBV implication in TL is possible but not common.

### III-B-2/Enterovirus

A patient with autoimmune thyroiditis had a transitory recurrence of her goiter during pregnancy with TPOAb becoming strongly positive. Six months post partum she had a subacute thyroiditis. Serology established the diagnosis of viral thyroiditis due to a Coxsackie-B virus. Two months later the goiter showed further growth, in association with cervical lymphadenopathy and an enlarged left parotid gland. Histology revealed a primary thyroid lymphoma.

### III-B-3/Human T Lymphotropic Virus (HTLV1)

Thyroid non-Hodgkin's lymphoma in an area in which adult T-cell leukemia/lymphoma (ATL) is not endemic is exclusively B-cell derived. A study was carried out to examine whether thyroid non-Hodgkin's lymphoma in an area in which ATL is endemic is also exclusively of B-cell type. Eight cases with thyroid non-Hodgkin's lymphoma admitted to the hospital situated in an ATL-endemic area were studied. Immunophenotypic study revealed all but one case to be of B-cell nature The T-cell type lymphoma case also had antibodies against HTLV-1 in the serum [134].

### III-B-4/Viral hepatitis C (HCV)

Lymphomas are frequent in HCV-infected patients but no thyroid lymphoma has been reported in these patients[107].

### III-B-5/Human Immunodeficiency Virus (HIV)

Two cases of thyroid lymphoma have been described in HIV-infected patients. The first is a 31-year old woman with acquired immunodeficiency syndrome (AIDS) who presented a severe thyrotoxicosis and a markedly enlarged, diffuse, tender goiter. The patient died within days of her presentation. At autopsy, near-complete replacement of the thyroid gland with anaplastic large cell lymphoma was found, without coexisting infectious or autoimmune processes in the gland [135]. The second case was a child with vertical transmission-acquired HIV, presenting with lymphomatous infiltration of the thyroid gland at diagnosis [136].

## Conclusion

Identifying etiological infections in human disease is difficult. Besides the fact that organ tissue is not always available for direct study, the interpretation of virological data must be cautious. The presence of antibodies directed towards a virus does not prove that this pathogen is responsible for the disease, especially when the agent is common in the general population. On the other hand, the absence of viral markers at the onset of the disease does not refute the viral hypothesis. Indeed the triggering infection can take place many years previously. A triggering virus can be cleared from the body without any virological trace except the presence of specific antibodies. It is relevant to look for viral agents in tissues in which they can persist without systemic manifestation. Direct evidence of the presence of viruses or their components in the organ are available for retroviruses (HFV) and mumps in subacute thyroiditis, for retroviruses (HTLV-1, HFV, HIV and SV40) in Graves's disease and for HTLV-1, enterovirus, rubella, mumps virus, HSV, EBV and parvovirus in Hashimoto's thyroiditis. However, it remains to determine whether they are responsible for thyroid diseases or whether they are just innocent bystanders.

A viral disease is the result of an interaction between a virus and the host, in which the genetic background plays a role. Therefore, it cannot be excluded that a virus plays a role in a disease even though most infected individuals do not show any sign of disease.

Further studies are needed to clarify the relationship between viruses and thyroid diseases, in order to develop new strategies for their prevention and/or the treatment.

## Competing interests

The authors declare that they have no competing interests.

## Authors' contributions

RD and DH conceived and wrote the review. Authors read and approved the final manuscript.
